# Supplementary material for: The structure, centrality, and scale of urban street networks: Cases from Pre-Industrial Afro-Eurasia
Source: PLoS One. 2021 Nov 11;16(11):e0259680. doi: 10.1371/journal.pone.0259680 (PMC8585513; doi:10.1371/journal.pone.0259680)
Supplement: S1 File — (DOCX) [file pone.0259680.s001.docx]

# Supporting information

Data and code for this work can be downloaded here: https://doi.org/[10.5522/04/15191601](https://doi.org/10.5522/04/15191601)
