# Supplementary material for: The structure, centrality, and scale of urban street networks: Cases from Pre-Industrial Afro-Eurasia
Source: PLoS One. 2021 Nov 11;16(11):e0259680. doi: 10.1371/journal.pone.0259680 (PMC8585513; doi:10.1371/journal.pone.0259680)
Supplement: Attachment — Submitted filename: Response_To_Reviewers.docx [file pone.0259680.s003.docx]

Dear Riccardo,

We very much appreciate this review and have accepted nearly all the comments indicated to us. We are happy to revise and provide the following list of comments, indicated in red for our response to specific editorial or review comments, in this letter. We also indicate the letter provided to us as a reference and our comments are embedded. Where we did not address a given concern, we indicate why and provide justification. However, in general, we have accepted comments given to us and address them. Thank you again and we appreciate this review.

Comments:

PONE-D-21-15963
The structure, centrality, and scale of urban street networks: Cases from Pre-Industrial Afro-Eurasia
PLOS ONE

Dear Dr. Altaweel,

Thank you for submitting your manuscript to PLOS ONE. After careful consideration, we feel that it has merit but does not fully meet PLOS ONE’s publication criteria as it currently stands. Therefore, we invite you to submit a revised version of the manuscript that addresses the points raised during the review process.

We have followed the editorial comments and reviewer comments. We hope these are adequately addressed. We indicate how we addressed points below under each point indicated.

In particular, please address with attention and details the comments requiring further comparisons on the different indicators presented, that might be presenting similar, correlated information, and the clarification of the manuscript's text in some of its passages.

Please submit your revised manuscript by Sep 30 2021 11:59PM. If you will need more time than this to complete your revisions, please reply to this message or contact the journal office at plosone@plos.org. When you're ready to submit your revision, log on to https://www.editorialmanager.com/pone/ and select the 'Submissions Needing Revision' folder to locate your manuscript file.

Please include the following items when submitting your revised manuscript:

- A rebuttal letter that responds to each point raised by the academic editor and reviewer(s). You should upload this letter as a separate file labeled 'Response to Reviewers'.
- A marked-up copy of your manuscript that highlights changes made to the original version. You should upload this as a separate file labeled 'Revised Manuscript with Track Changes'.
- An unmarked version of your revised paper without tracked changes. You should upload this as a separate file labeled 'Manuscript'.

If you would like to make changes to your financial disclosure, please include your updated statement in your cover letter. Guidelines for resubmitting your figure files are available below the reviewer comments at the end of this letter.

We changed the financial disclosure in the Cover Letter, indicating the funding source of this work.

If applicable, we recommend that you deposit your laboratory protocols in protocols.io to enhance the reproducibility of your results. Protocols.io assigns your protocol its own identifier (DOI) so that it can be cited independently in the future. For instructions see: [http://journals.plos.org/plosone/s/submission-guidelines#loc-laboratory-protocols](https://eur01.safelinks.protection.outlook.com/?url=http%3A%2F%2Fjournals.plos.org%2Fplosone%2Fs%2Fsubmission-guidelines%23loc-laboratory-protocols&data=04|01|m.altaweel@ucl.ac.uk|95bbc97ba62c4bc875fc08d960d3593a|1faf88fea9984c5b93c9210a11d9a5c2|0|0|637647284786431731|Unknown|TWFpbGZsb3d8eyJWIjoiMC4wLjAwMDAiLCJQIjoiV2luMzIiLCJBTiI6Ik1haWwiLCJXVCI6Mn0%3D|1000&sdata=C0zv5qQvX8MAKalFadgYFwV4S5a8Xmc1r1fRRyDP3s0%3D&reserved=0). Additionally, PLOS ONE offers an option for publishing peer-reviewed Lab Protocol articles, which describe protocols hosted on protocols.io. Read more information on sharing protocols at [https://plos.org/protocols?utm_medium=editorial-email&utm_source=authorletters&utm_campaign=protocols](https://eur01.safelinks.protection.outlook.com/?url=https%3A%2F%2Fplos.org%2Fprotocols%3Futm_medium%3Deditorial-email%26utm_source%3Dauthorletters%26utm_campaign%3Dprotocols&data=04|01|m.altaweel@ucl.ac.uk|95bbc97ba62c4bc875fc08d960d3593a|1faf88fea9984c5b93c9210a11d9a5c2|0|0|637647284786441729|Unknown|TWFpbGZsb3d8eyJWIjoiMC4wLjAwMDAiLCJQIjoiV2luMzIiLCJBTiI6Ik1haWwiLCJXVCI6Mn0%3D|1000&sdata=J8TgJ9OpPvHdb7ccpGLsdNjysVFzDdZ7%2Fevf4w2BgIg%3D&reserved=0).

We look forward to receiving your revised manuscript.

Kind regards,

Riccardo Gallotti
Academic Editor
PLOS ONE

Journal requirements:

When submitting your revision, we need you to address these additional requirements.

1. Please ensure that your manuscript meets PLOS ONE's style requirements, including those for file naming. The PLOS ONE style templates can be found at https://journals.plos.org/plosone/s/file?id=wjVg/PLOSOne_formatting_sample_main_body.pdf and [https://journals.plos.org/plosone/s/file?id=ba62/PLOSOne_formatting_sample_title_authors_affiliations.pdf](https://eur01.safelinks.protection.outlook.com/?url=https%3A%2F%2Fjournals.plos.org%2Fplosone%2Fs%2Ffile%3Fid%3Dba62%2FPLOSOne_formatting_sample_title_authors_affiliations.pdf&data=04|01|m.altaweel@ucl.ac.uk|95bbc97ba62c4bc875fc08d960d3593a|1faf88fea9984c5b93c9210a11d9a5c2|0|0|637647284786441729|Unknown|TWFpbGZsb3d8eyJWIjoiMC4wLjAwMDAiLCJQIjoiV2luMzIiLCJBTiI6Ik1haWwiLCJXVCI6Mn0%3D|1000&sdata=5sarvT1AuNXyKZJhtznir%2F0YtsgXMCYkkPqZWqv7gug%3D&reserved=0).

We checked and followed the guidelines provided.

2. In your manuscript, please provide additional information regarding the specimens used in your study. Ensure that you have reported specimen numbers and complete repository information, including museum name and geographic location.

We provided a .shp file for the location information. The data are all referenced, with the relevant shapefiles provided which derived from the maps referenced in the Supplementary Informationl repository provided. All data are in the Supplementary Information link provided.

If permits were required, please ensure that you have provided details for all permits that were obtained, including the full name of the issuing authority, and add the following statement:

'All necessary permits were obtained for the described study, which complied with all relevant regulations.'

If no permits were required, please include the following statement:

'No permits were required for the described study, which complied with all relevant regulations.'

For more information on PLOS ONE's requirements for paleontology and archaeology research, see [https://journals.plos.org/plosone/s/submission-guidelines#loc-paleontology-and-archaeology-research](https://eur01.safelinks.protection.outlook.com/?url=https%3A%2F%2Fjournals.plos.org%2Fplosone%2Fs%2Fsubmission-guidelines%23loc-paleontology-and-archaeology-research&data=04|01|m.altaweel@ucl.ac.uk|95bbc97ba62c4bc875fc08d960d3593a|1faf88fea9984c5b93c9210a11d9a5c2|0|0|637647284786451728|Unknown|TWFpbGZsb3d8eyJWIjoiMC4wLjAwMDAiLCJQIjoiV2luMzIiLCJBTiI6Ik1haWwiLCJXVCI6Mn0%3D|1000&sdata=RUpYKenlLg0YEt%2FjdeG0TSK2gLvdJnj96tZsTDw2wJE%3D&reserved=0).

We have now provided an indication in the article in the Case Studies section discussing that the information used required no permissions as they were all public and/or published data.

3. We note that the grant information you provided in the ‘Funding Information’ and ‘Financial Disclosure’ sections do not match.

When you resubmit, please ensure that you provide the correct grant numbers for the awards you received for your study in the ‘Funding Information’ section.

The Center of Advanced Studies-Schwerpunkt (Siedlungen zwischen Diversität und Homogenität) from the University of Munich (LMU) funded this work through a fellowship. There is no number of the award as it funded a sabbatical leave when the work was developed. We indicated this in the Acknowledgments and Funding Information now. We hope it is clear.

4. Please note that in order to use the direct billing option the corresponding author must be affiliated with the chosen institute. Please either amend your manuscript to change the affiliation or corresponding author, or email us at plosone@plos.org with a request to remove this option.

The billing should go to UCL, which is the first author’s affiliation. They have an agreement/payment setup with PlosOne for articles to be published. In the revision, we do not see where to amend this or indicate?

5. We note that Figures 1-3 in your submission contain [map/satellite] images which may be copyrighted. All PLOS content is published under the Creative Commons Attribution License (CC BY 4.0), which means that the manuscript, images, and Supporting Information files will be freely available online, and any third party is permitted to access, download, copy, distribute, and use these materials in any way, even commercially, with proper attribution. For these reasons, we cannot publish previously copyrighted maps or satellite images created using proprietary data, such as Google software (Google Maps, Street View, and Earth). For more information, see our copyright guidelines: <http://journals.plos.org/plosone/s/licenses-and-copyright>.

We have removed the underlying maps for Figures 1 and 3. We have now added Natural Earth data for Figure 1’s underlying data and the credit is given in the caption. For Figure 2, we now have permission to use the image and this is indicated in the caption and uploaded permission document.

We require you to either (1) present written permission from the copyright holder to publish these figures specifically under the CC BY 4.0 license, or (2) remove the figures from your submission:
 
a. You may seek permission from the original copyright holder of Figures 1-3 to publish the content specifically under the CC BY 4.0 license. 
 
We recommend that you contact the original copyright holder with the Content Permission Form (http://journals.plos.org/plosone/s/file?id=7c09/content-permission-form.pdf) and the following text:
“I request permission for the open-access journal PLOS ONE to publish XXX under the Creative Commons Attribution License (CCAL) CC BY 4.0 (http://creativecommons.org/licenses/by/4.0/). Please be aware that this license allows unrestricted use and distribution, even commercially, by third parties. Please reply and provide explicit written permission to publish XXX under a CC BY license and complete the attached form.”
 
Please upload the completed Content Permission Form or other proof of granted permissions as an "Other" file with your submission.
 
In the figure caption of the copyrighted figure, please include the following text: “Reprinted from [ref] under a CC BY license, with permission from [name of publisher], original copyright [original copyright year].”
 
b.  If you are unable to obtain permission from the original copyright holder to publish these figures under the CC BY 4.0 license or if the copyright holder’s requirements are incompatible with the CC BY 4.0 license, please either i) remove the figure or ii) supply a replacement figure that complies with the CC BY 4.0 license. Please check copyright information on all replacement figures and update the figure caption with source information. If applicable, please specify in the figure caption text when a figure is similar but not identical to the original image and is therefore for illustrative purposes only.
The following resources for replacing copyrighted map figures may be helpful:
 
USGS National Map Viewer (public domain): http://viewer.nationalmap.gov/viewer/
The Gateway to Astronaut Photography of Earth (public domain): http://eol.jsc.nasa.gov/sseop/clickmap/
Maps at the CIA (public domain): https://www.cia.gov/library/publications/the-world-factbook/index.html and https://www.cia.gov/library/publications/cia-maps-publications/index.html
NASA Earth Observatory (public domain): http://earthobservatory.nasa.gov/
Landsat: http://landsat.visibleearth.nasa.gov/
USGS EROS (Earth Resources Observatory and Science (EROS) Center) (public domain): http://eros.usgs.gov/#
Natural Earth (public domain): http://www.naturalearthdata.com/

Please review your reference list to ensure that it is complete and correct. If you have cited papers that have been retracted, please include the rationale for doing so in the manuscript text, or remove these references and replace them with relevant current references. Any changes to the reference list should be mentioned in the rebuttal letter that accompanies your revised manuscript. If you need to cite a retracted article, indicate the article’s retracted status in the References list and also include a citation and full reference for the retraction notice.

We have added four new references. These references are numbed 37-39 and 59 in the references given.

Additional Editor Comments (if provided):


[Note: HTML markup is below. Please do not edit.]

Reviewers' comments:

Reviewer's Responses to Questions

**Comments to the Author**

1. Is the manuscript technically sound, and do the data support the conclusions?

The manuscript must describe a technically sound piece of scientific research with data that supports the conclusions. Experiments must have been conducted rigorously, with appropriate controls, replication, and sample sizes. The conclusions must be drawn appropriately based on the data presented.

Reviewer #1: Yes

Reviewer #2: Yes

2. Has the statistical analysis been performed appropriately and rigorously?

Reviewer #1: Yes

Reviewer #2: Yes

3. Have the authors made all data underlying the findings in their manuscript fully available?

The [PLOS Data policy](https://eur01.safelinks.protection.outlook.com/?url=http%3A%2F%2Fwww.plosone.org%2Fstatic%2Fpolicies.action%23sharing&data=04|01|m.altaweel@ucl.ac.uk|95bbc97ba62c4bc875fc08d960d3593a|1faf88fea9984c5b93c9210a11d9a5c2|0|0|637647284786461715|Unknown|TWFpbGZsb3d8eyJWIjoiMC4wLjAwMDAiLCJQIjoiV2luMzIiLCJBTiI6Ik1haWwiLCJXVCI6Mn0%3D|1000&sdata=o1M%2B6xll4zpHPsJEc7P4ftKbw6iNg8pJDYFkxAphFmA%3D&reserved=0) requires authors to make all data underlying the findings described in their manuscript fully available without restriction, with rare exception (please refer to the Data Availability Statement in the manuscript PDF file). The data should be provided as part of the manuscript or its supporting information, or deposited to a public repository. For example, in addition to summary statistics, the data points behind means, medians and variance measures should be available. If there are restrictions on publicly sharing data—e.g. participant privacy or use of data from a third party—those must be specified.

Reviewer #1: Yes

Reviewer #2: Yes

4. Is the manuscript presented in an intelligible fashion and written in standard English?

PLOS ONE does not copyedit accepted manuscripts, so the language in submitted articles must be clear, correct, and unambiguous. Any typographical or grammatical errors should be corrected at revision, so please note any specific errors here.

Reviewer #1: Yes

Reviewer #2: Yes

5. Review Comments to the Author

Please use the space provided to explain your answers to the questions above. You may also include additional comments for the author, including concerns about dual publication, research ethics, or publication ethics. (Please upload your review as an attachment if it exceeds 20,000 characters)

Reviewer #1: Title
The structure, centrality, and scale of urban street networks: Cases from Pre-Industrial Afro-Eurasia

This ms seeks to describe scaling relationships and Centrality of urban street networks across a large set of pre-intdustrial and ancient cities in Eurasia.
I enjoyed reading this ms. It is well written and scientifically sound. Methods are wel described.

This is a great contribution to the literature building on specific findings of urban scaling.

I have some concerns regarding the definition of hybrid/organic cities, while it seem intuitive, it would be helpfull to see which cities have been classified as such. A suggestion for this would be to include such information in Figure 1 perhaps.

We have now put which cities were classified as ‘orthogonal’, ‘organic’ and ‘hybrid’ in Figure 1. We also provided definitions of the categories used in the beginning of the material and methods section. The data in the Supplementary Material also indicates what urban locations are what type.

My next concern regards the analyses of density plots. Some Centrality metrics show bimodal distribution. Why is that? Could this be related to my previous point? Particularly, Harmonic, Degree, Straightness, Katz and Betweenness to some degree. This is worth mentioning in the discussion.

This is now discussed in the discussion section. It appears that this is the case mostly for orthogonal, which we believe relates to the fact that such cities promote somewhat higher connectivity across wider areas of urban layouts than organic or even hybrid sites. So what is going on is that the distributions are more spread, and somewhat bimodal in places, relative to organic/hybrid urban locations. We explain this in the discussion section.

Reviewer #2: General comment
The paper shows results of common centrality properties for different types of cities found in different regions as well as different periods.
It's a very interesting idea to investigate scaling relationships for centrality measures in different types of cities. The methodology and the outputs are also very interesting with a significant added value within the geo-archeological fieldwork.
The results demonstrated different centrality distributions and different scaling relationships between planned and organic cities.

Minor global comments:

- There are many indicators computed and presented. One could argue that some of them may bring the same kind of information. It could be interesting to show that they are not giving correlated results.

We provide a statistical comparison in the results. We indicate that at a p-value<0.01 significance level, all the distributions (between and within each category) are significantly different. We were also a little unclear about this comment but we hope we understood it and addressed it appropriately. We also qualitatively describe the distributions in the results and discussion.

- The text is overall very well structured and clear, but few times sentences are quite long, with a complex structure, and we miss the point. Those specific sentences are detailed in the next paragraph “detailed comments.”

We appreciate these suggestions and we have mostly followed them.


Detailed comments:

Abstract
l.20 – l.22: The sentence is hard to read. It would be better to reformulate.

This is now changed and the sentence is edited to be more clear.

Introduction
l. 59: The term of “urban area” could be better defined.

This is now defined (briefly) in the introduction.

l. 69: repetition of “assess”

Many of these are removed and other words are now used.

l.79: “…and centrality and urban area.” You can use a comma to avoid repeating “and” and make the sentence clearer.

We revised to make it clearer.

l.80: We have the information about the layouts after but few words more about it in this part could be relevant.

Ok a fuller definition is now provided in the material and methods section, but an indication of the types of settlements evaluated is now provided in the introduction as well.

l. 97: repetition of “how”

This is now edited.

l.112 – l.116: The sentence is too complex. It would be better to simplify by writing one sentence for one idea at a time.

This is now edited.

Background
The background section presents the scientific context of the paper that was already addressed in the introduction section without being detailed. It seems possible to avoid some redundancies between the two sections.

We removed material that was repetitive or elaborated on points in the background that were indicated in the introduction.

Urban Scaling
In this paragraph the word “demonstrate” is used 12 times! It would be better to reformulate. (Especially the sentence l. 131 to 134).
l. 134 : typo : [7,30)

Good suggestions! We have reformed this section and followed the suggestions.

Materials and Methods
l. 173-174: The classification in “orthogonal” or “organic” would gain to be developed

We have now added a paragraph at the beginning of the section to clarify this.

l.229 – l.233: The sentence is unclear (too long).

This is now edited.

l.247: I would suggest adding one sentence to explain the 30 degree threshold.

We have now added an explanation. We indicate that the degree is sharp enough where it represents an intersection.

Results

- In figures 4 and 5, x and y axes are not with the same scale between plots of the same indicator for each types, which make their comparison harder.

Yes, we did try to make the scales the same but due to distribution differences it was not feasible since some of the information, particularly in figure 5, would be hard to notice or see. We have now stated this in the results to make this clear.

- In figure 5 the legend mention a “dashed lines showing median values” which is not present on the plots.

This is now changed so that there is a dashed line for Figure 5.

- In figure 6, same problem of scale between 6a and 6c on the one hand, and 6b and 6d on the other.

Yes this is also due to variations in where values concentrated, which was different between 6a-6d. We tried to standardize but it made some of the values hard to see, which is why we made the outputs then with somewhat different scales. The trends are hopefully clear.

- Figures 7 and 8: Why it is not the same number of periods? Is it related to data availability? It would be better to explain why the results are comparable while the inputs considered are not the same for the two different street layout type.

Yes, there are variations in how much data for each period and also the types of cities (orthogonal vs. organic/hybrid). However, some comparability is evident since we get some similar results. We explain this now in the results section for the figures.

6. PLOS authors have the option to publish the peer review history of their article ([what does this mean?](https://eur01.safelinks.protection.outlook.com/?url=https%3A%2F%2Fjournals.plos.org%2Fplosone%2Fs%2Feditorial-and-peer-review-process%23loc-peer-review-history&data=04|01|m.altaweel@ucl.ac.uk|95bbc97ba62c4bc875fc08d960d3593a|1faf88fea9984c5b93c9210a11d9a5c2|0|0|637647284786461715|Unknown|TWFpbGZsb3d8eyJWIjoiMC4wLjAwMDAiLCJQIjoiV2luMzIiLCJBTiI6Ik1haWwiLCJXVCI6Mn0%3D|1000&sdata=YbtY3TUe03bOqohpBJ0JZOuXmk83czoJm3djhTfGCes%3D&reserved=0)). If published, this will include your full peer review and any attached files.

Yes this is fine to publish the history of the peer review.

If you choose “no”, your identity will remain anonymous but your review may still be made public.


**Do you want your identity to be public for this peer review?** For information about this choice, including consent withdrawal, please see our [Privacy Policy](https://eur01.safelinks.protection.outlook.com/?url=https%3A%2F%2Fwww.plos.org%2Fprivacy-policy&data=04|01|m.altaweel@ucl.ac.uk|95bbc97ba62c4bc875fc08d960d3593a|1faf88fea9984c5b93c9210a11d9a5c2|0|0|637647284786471706|Unknown|TWFpbGZsb3d8eyJWIjoiMC4wLjAwMDAiLCJQIjoiV2luMzIiLCJBTiI6Ik1haWwiLCJXVCI6Mn0%3D|1000&sdata=2q8B0XLZGJ4FmTj7Cr0w5RUJgR%2BGfJu60xcG%2F%2Fy886c%3D&reserved=0).

Reviewer #1: **Yes:** Horacio Samaniego

Reviewer #2: **Yes:** Claire Lagesse

[NOTE: If reviewer comments were submitted as an attachment file, they will be attached to this email and accessible via the submission site. Please log into your account, locate the manuscript record, and check for the action link "View Attachments". If this link does not appear, there are no attachment files.]

While revising your submission, please upload your figure files to the Preflight Analysis and Conversion Engine (PACE) digital diagnostic tool, [https://pacev2.apexcovantage.com/](https://eur01.safelinks.protection.outlook.com/?url=https%3A%2F%2Fpacev2.apexcovantage.com%2F&data=04|01|m.altaweel@ucl.ac.uk|95bbc97ba62c4bc875fc08d960d3593a|1faf88fea9984c5b93c9210a11d9a5c2|0|0|637647284786481709|Unknown|TWFpbGZsb3d8eyJWIjoiMC4wLjAwMDAiLCJQIjoiV2luMzIiLCJBTiI6Ik1haWwiLCJXVCI6Mn0%3D|1000&sdata=rG4EKx0Cfp2pstcyUgFpVg%2Bm0w4LtdL7OYEK%2FqlfNYo%3D&reserved=0). PACE helps ensure that figures meet PLOS requirements. To use PACE, you must first register as a user. Registration is free. Then, login and navigate to the UPLOAD tab, where you will find detailed instructions on how to use the tool. If you encounter any issues or have any questions when using PACE, please email PLOS at [figures@plos.org](mailto:figures@plos.org). Please note that Supporting Information files do not need this step.

We have checked our figures using this site and all of them appear to be of good quality and sufficient. Also, we noticed in the notes in the pdf of the manuscript it stated that the colors were hard to read for some figures. However, when looking at the high quality tiff files, the colors look very clearly different. We think that the pdf diminished the quality of the figures, which may explain why the colors are hard to differentiate.
